# Conduct disorder in girls: neighborhoods, family characteristics, and parenting behaviors

**DOI:** 10.1186/1753-2000-2-28

**Published:** 2008-10-06

**Authors:** Kathleen Pajer, Stefanie Stein, Karin Tritt, Chien-Ni Chang, Wei Wang, William Gardner

**Affiliations:** 1 The Ohio State University College of Medicine, Department of Pediatrics, Research Institute at Nationwide Children's Hospital, Columbus, OH, USA; 2 Department of Psychosomatic Medicine, University Clinic, Regensburg, Germany

## Abstract

**Background:**

Little is known about the social context of girls with conduct disorder (CD), a question of increasing importance to clinicians and researchers. The purpose of this study was to examine the associations between three social context domains (neighborhood, family characteristics, and parenting behaviors) and CD in adolescent girls, additionally testing for race moderation effects. We predicted that disadvantaged neighborhoods, family characteristics such as parental marital status, and parenting behaviors such as negative discipline would characterize girls with CD. We also hypothesized that parenting behaviors would mediate the associations between neighborhood and family characteristics and CD.

**Methods:**

We recruited 93 15–17 year-old girls from the community and used a structured psychiatric interview to assign participants to a CD group (n = 52) or a demographically matched group with no psychiatric disorder (n = 41). Each girl and parent also filled out questionnaires about neighborhood, family characteristics, and parenting behaviors.

**Results:**

Neighborhood quality was not associated with CD in girls. Some family characteristics (parental antisociality) and parenting behaviors (levels of family activities and negative discipline) were characteristic of girls with CD, but notll. There was no moderation by race. Our hypothesis that the association between family characteristics and CD would be mediated by parenting behaviors was not supported.

**Conclusion:**

This study expanded upon previous research by investigating multiple social context domains in girls with CD and by selecting a comparison group who were not different in age, social class, or race. When these factors are thus controlled, CD in adolescent girls is not significantly associated with neighborhood, but is associated with some family characteristics and some types of parental behaviors. However, the mechanisms underlying these relationships need to be further investigated. We discuss possible explanations for our findings and suggest directions for future research.

## Introduction

The diagnosis of conduct disorder (CD) identifies youths who persistently exhibit behaviors that violate rules and the rights of others [1]. CD is less common in girls than it is in boys, but the prevalence is not trivial. Up to 10% of adolescent girls in the U.S. meet criteria for CD [2-6] and epidemiological data from the UK and Canada indicate that approximately 3.5% of adolescent girls have CD [7-9]. CD is the second most common psychiatric diagnosis in adolescent girls [10,11]. Moreover, many girls with CD have significant problems in adulthood, including the development of mental and physical health problems and difficulties in parenting [12-17].

Despite these findings, there is a paucity of data on girls with CD [18,19]. Much of the information about "CD in girls" is actually derived from studies of boys with CD, girls with delinquency, or studies of girls' problem behaviors. The use of these types of studies presents difficulties for clinicians trying to determine what treatments are effective specifically for girls with CD and for researchers trying to elucidate social or biological mechanisms in the development of CD in females. Data from boys do not necessarily generalize to girls. Gender differences have been reported for CD symptom presentation, prevalence rates, comorbidities, antecedents, correlates, and prognosis [11,20,21].

Similar issues arise with drawing conclusions from studies about delinquency. Delinquency and CD are not the same; committing illegal acts (delinquency) is only one of several components in the CD diagnosis [1]. Moreover, in boys, CD and delinquency predict separate trajectories of adult dysfunction [22,23]. Many delinquent girls do not meet criteria for CD and vice versa. For example, only 39% of delinquent girls in a large sample of detainees met criteria for CD [17]. Conversely, only 20% of the girls with *any *type of psychiatric disorder were arrested during in a population study of youths receiving mental health services [24].

The use of studies about problem disorders also presents difficulties. The definition "problem behaviors" varies widely between studies, e.g., adolescent pregnancy, substance abuse, or symptom scores of all the disruptive behavior disorders. Girls with these characteristics may be more likely to have CD, but problem behaviors are clearly not the same as a diagnosis of CD. In summary, studies about boys with CD, or girls with delinquency, or girls with problem behaviors can stimulate research questions about girls with CD. But, to understand the pathogenesis of the syndrome in females and develop effective treatment, we need to actually examine girls with CD.

One of the questions for which there are particularly few data on girls with CD is how social context shapes the development of the disorder. The issue is particularly salient at this time because of renewed interest in how social context impacts child and adolescent psychiatry and developmental psychopathology [25-29]. Social context is also well-accepted as a critical element in the development of CD in boys and in delinquency [30,31]. Because there is some overlap between girls with CD and these populations, it is highly likely that social context is also important in understanding girls' CD. However, significant sex differences exist in how social context affects delinquency [32], suggesting that we cannot assume that the relationships that social context has with CD in girls will be the same as found in boys with CD or girl with delinquency. Thus, studies are needed that examine social context in girls who have CD.

To our knowledge, only three studies have done this. Johnson and O'Leary found that two family characteristics (maternal and paternal aggressiveness) were associated with CD in a community sample of 43 9–11-year-old girls (25 with research diagnosis of CD, 17 without CD) [33]. In contrast to data on boys, this study did not find a significant association between parenting behaviors and CD in girls, but this may have been due to their data collection method (asking parents how they would parent their daughters in hypothetical situations) or the small sample size. In a large birth cohort study, family characteristics (social class, single mother status, young mother, multiple caregivers, parental antisocial behavior) and one parenting behavior (harsher discipline) were associated with the total number of CD and delinquent behaviors in adolescent girls [34]. In Finland, three family characteristics (nonspecified parental psychopathology, not living with both biological parents, low socioeconomic status) and one parenting behavior (physical abuse) were associated with the diagnosis of CD in adolescent girls who were on a psychiatric inpatient unit [35].

Taken together, these three studies indicate that some social context factors are associated with CD in girls, but also suggest that the associations may be different from those reported in boys with CD. There are several important limitations to the use of these studies: the samples were comprised of white girls, a small number of social context indicator variables were used, only one or two domains of social context were examined, and no investigations of between-domain associations were conducted. These problems are frequently found in research on social context and psychiatric disorders, according to a critical review conducted by The MacArthur Network on Psychopathology and Development [28]. The review concluded that social contexts must be viewed as separate, but nested and multidimensional and that research is needed to study associations between social contexts. Based on these suggestions, the next step in understanding the social context of girls with CD would be to use a more comprehensive set of indicator variables, examine multiple social context domains, and study inter-domain relationships with CD. But beyond attempting to replicate the work from the three earlier studies on girls with CD, how should we select the variables and domains to study?

As discussed above, studies of social context that examine boys, delinquent girls, or youths with variously defined problem behaviors are not sufficient for drawing conclusions about girls with CD. But they are useful for shaping research questions. These sources of data indicate that factors such as growing up in a disadvantaged neighborhood, having a single mother, and some types of parenting behaviors (e.g., harsh or physical discipline) are frequently associated with, or predictive of adolescent antisocial behavior [18,36-43]. Several studies have further reported that associations between adolescent antisocial behaviors and neighborhood, social class, or family structure are mediated by parenting behaviors [44-49]. Some of these relationships may differ by race, although the findings are contradictory. Two studies reported that parenting behaviors did not predict antisocial behavior in blacks, but did so in whites [50,51], while no race differences were found in two other studies [45,52]. In conclusion, numerous studies indicate that neighborhood quality, family characteristics, and parenting behaviors are all important social context domains in the development of CD in boys, delinquency in girls, or various types of problem behaviors in girls; these associations may be moderated by race. Several studies have demonstrated that in girls, social context levels involving more intimate contact (e.g., parenting behaviors) mediate the relationships between those that are less relationship dependent (e.g., family characteristics or neighborhood). Therefore, the purpose of our study was to determine if these findings about social context could be replicated in adolescent girls with diagnoses of CD. We used a sample of community girls with CD and compared them to a demographically similar group of girls without any psychopathology to answer the following five questions about social context:

1) Is neighborhood disadvantage significantly associated with CD?

2) Are family characteristics (e.g., family structure and parental psychopathology) correlated with CD?

3) Do parenting behaviors differ between girls with and without CD?

4) Does race (white or African-American) moderate any of the above associations?

5) Are the associations between neighborhood or family characteristics and CD in girls mediated by parenting behaviors?

We predicted that girls with CD, compared to girls without any psychiatric disorder, would be characterized by: living in more disadvantaged neighborhoods, having families with younger mothers, higher rates of unmarried parents, and higher rates of parental psychopathology, and being exposed to more negative parenting behaviors (negative discipline) and less supervision and consistency in discipline. We also expected race to moderate these associations, i.e., that some or all of these associations with CD would only be found in the white girls.

## Methods

### Recruitment and sample selection

Subjects comprised the baseline sample for a longitudinal study about young adult outcomes of CD in adolescent girls. Participants were recruited through newspaper ads that asked: "Do you know a 15–17 year-old girl with behaviors such as truancy, fighting, stealing, and lying?" Ads also stated that girls without these behaviors were needed for the study. When a girl or parent called in response to an ad, the study was explained and the caller screened for participant eligibility. The staff member also spoke with either the parent/guardian (if a girl made the first contact) or the girl (if the parent called first) to explain the study. If both wanted to participate, an appointment was made for the girl and her parent or guardian (most were mothers).

A structured psychiatric interview (see below) was used to categorize the girls as having CD or having no psychiatric disorder (NC group). Exclusion criteria for both groups were: age outside the 15–17-year range, head trauma with loss of consciousness for ≥ 15 minutes, serious medical illness, IQ < 65, history of psychosis, or pubertal development less than Tanner Stage V. Medical history and Tanner staging were exclusion criteria necessary for the psychoneuroendocrinological portion of the study and exclusion of girls with low IQ or head trauma was needed for the neuropsychological protocol. The results from these portions of the study are reported elsewhere [53-55]. Of the 151 parent-youth dyads interviewed, 93 girls (52 CD and 41 NC) were found to be eligible and agreed to participate (no girl refused).

### Protocol

When participants arrived for the interview, staff explained the study in detail and answered questions. Written, informed assent/consent was obtained from each girl and parent or guardian. Interviews were conducted concurrently, but separately, with each member of the dyad. At the end of the interview, the girl and adult received $20 and money for parking or bus fare. The study was approved by the Institutional Review Boards of Allegheny University of the Health Sciences and the University of Pittsburgh School of Medicine.

### Constructs and instruments

#### 1. Psychiatric diagnosis

Psychiatric diagnoses were determined with the computerized version of the Diagnostic Interview Schedule for Children (C-DISC), Parent and Youth Versions [56,57]. Interviewers had bachelor or post-graduate degrees and were trained by one of the authors (KP), who had been trained by the developers of the DISC. The validity of using the age of onset and aggression criteria to diagnose CD in girls has been questioned [11,18,58]. Therefore, we slightly modified the DISC algorithm for CD. We required that antisocial behaviors be present for at least one year prior to the interview, but dropped the criterion that required age of onset before 13 years of age. In addition, the question about fighting was changed from "Do you often start fights?" to "Do you often get into fights?"

A psychiatric diagnosis was assigned on the basis of the data from the adolescent *or *parent. At this age, it is difficult to know who is the better informant [59]. Some adolescents may tell their parents about their activities and feelings and others may not. Moreover, both adolescents and parents may exaggerate or minimize symptoms. Therefore, we took all information as equally valid.

#### 2. IQ (exclusion criterion)

IQ was measured by the Kaufman Brief Intelligence Test (K-BIT) [60]. This instrument produces a score for the verbal and nonverbal components of intelligence, as well as a composite score.

#### 3. Neighborhood

Each girl's residence was matched via zip code to neighborhood data from the U.S. Census Bureau (2000 Census) to determine neighborhood quality. The variables used for this study were percentages of: 1) housing units vacant or boarded up; 2) adults with less than a high school education; 3) unemployed adults; 4) families with income below the poverty level; 5) single mothers with children younger than 19 years. Because there are no studies about the association between neighborhoods and CD in girls, we based variable selection on previous studies of neighborhoods and delinquency [40,42,43].

#### 4. Family characteristics

This domain consisted of family structure variables and parental psychopathology. A structured interview was administered to the parent to obtain information about each girl's age, race, the parents' education and occupational levels, the marital status of the girl's biological parents and mother's age at the time of the girl's birth. Family social class was calculated with the four-factor Hollingshead Scale [61]. If two adults were living in the household and both contributed to the care of the girl, the social class was based on the highest category.

Data about biological parents' psychopathology were collected from the adult informants, using a variation of the Family History – Research Diagnostic Criteria (FH-RDC) [62]. The parent was shown cards on which the DSM-IV criteria for the following diagnoses were printed in lay terms: Major Depression, Antisocial Personality Disorder, Conduct Disorder, Drug and Alcohol Use Disorders (the general criteria were listed and then the individual substances were presented). After the research assistant read a diagnosis card with the parent and answered questions, the parent was asked whether each family member (e.g., mother, father, paternal grandmother) met criteria for the disorder. Only diagnoses in biological parents were used for this study.

The final variables used in this social context level were: 1) family social class; 3) age at which mother gave birth to girl; 4) marital status (never married vs. divorced, widowed, separated); 5) parental history of antisocial behavior; 6) parental history of depression, 7) parental history of substance use disorders (abuse or dependence).

#### 5. Parenting behaviors

Data for this social context level was collected with the parent and youth questionnaires used in the Pittsburgh Youth Study about the development of antisocial behavior in boys [63]; these instruments were based on the Oregon Social Learning Center theories of family management as related to antisocial behavior [64].

Information was collected about the following parenting behaviors: 1) family activities; 2) positive discipline; 3) negative discipline; 4) consistency of discipline; and 5) supervision. Questions about family activities asked about the frequency of specific types of family activities (e.g., "How often do you eat family dinners together?"). Items about positive discipline inquired about the frequencies of positive parenting behaviors (e.g. "How often do you/your parents tell her/you she's/you've done a good job?"); the format was similar for questions about negative discipline (e.g. "How often do you/your parents yell at her/you about what she/you did wrong?"). Consistency of discipline questions collected data on both positive and negative disciplinary techniques, (e.g., "When you/your parents promise her/you something good, how often do you/they stick to it?" and "When you/your parents ground her/you, how often do you/they carry it out?"). Supervision was evaluated with questions such as "How often do your parents know exactly where you are?" Answers for all parenting behaviors were obtained on a 4 point Likert scale ranging from "Never" to "Always" and the final scores for each was a simple sum with some reverse coding.

#### 6. Race

The race of the girl (African-American or white) was determined from an interview question asked of the adult informant.

### Data analysis

Data were checked for outliers and normality of distributions and log-transformed if normality was violated. Examination of patterns of missing data (less than 20% on any variable) showed that they were missing at random with respect to measured covariates. Multiple imputation was used to generate random scores to replace missing observations [65,66]. Five datasets with random replacements were generated using SAS Proc MI. These data sets were replicates, except for the randomly varying replacement scores. All statistical analyses were replicated across the five data sets [67,68]. To obtain our final statistical test results and *p *values, the five replicated test results were combined in one of two ways, depending on the type of variable. Proc MIANALYZE was used to combine results from analyses of continuous variables. Paul Allison's SAS macro  was used to combine the five *Chi*-square results for the categorical variables [69].

Multiple logistic regression was used to determine if CD was associated with neighborhood quality, family characteristics, or parenting behaviors (Questions 1–3). However, because each social context level contained many variables, it was necessary to reduce the number of variables in each domain and protect against a Type I error resulting from multiple comparisons. Therefore, before conducting the logistic regressions, we first used multivariate analysis of variables (MANOVA) to test whether CD girls differed from NC girls on vectors of social context levels or domains. If the *F *value for an overall MANOVA was statistically significant, *post hoc *analyses were used to examine the associations between CD and each of the individual variables included in the social context level. The Bonferroni correction was used in this process to reduce the risk of a Type I error. Only variables that remained significantly associated with CD in this two-step process were used as independent variables in the multiple logistic regressions equations.

To determine if race moderated any of the previous relationships between social context levels and CD (Question 4), we added race interaction factors to the logistic regression equations used in Questions 1–3.

We used the Barron and Kenny procedure [70] and the Sobel *z *statistic [71] to examine whether parenting behaviors mediated the associations between either neighborhood or family characteristics and CD (Question 5). Again, it was important to reduce the number of variables involved in the analyses, so we considered only those variables in the neighborhood, family characteristics, or parenting behaviors social context levels that remained significantly associated with CD after the two-step data reduction process described above.

## Results

### Participant characteristics

As can be seen in Table 1, there were no significant group differences in the mean age, proportions of girls in lower and higher social class categories, or proportions of white and African-American girls.

**Table 1:** Participant characteristics conduct disorder (CD) vs. no psychiatric disorder (NC)

**Variable**	**CD (n = 52)**	**NC (n = 41)**	**Statistic**	***p *value**
	**Mean (SD) or Percentage**			

**Age**	16.37 yrs(.83)	16.15 yrs(.85)	-1.25	0.211

**Race**				

White	59.6	73.2		
African-American	40.4	26.8	1.87	0.173

**Social Class**^1^				

High	11.5	26.8		
Low	88.5	73.2	3.59	0.064

^1 ^High: Categories 1 & 2; Low: Categories 3–5 on the Hollingshead Scale

Question 1: Is neighborhood disadvantage significantly associated with CD?

As displayed in Table 2, MANOVA did not indicate that the CD and NC groups were any different on the overall social context domain of neighborhood. Therefore, no further testing was conducted with this social context level.

**Table 2:** Neighborhood quality, family characteristics, and parenting behaviors conduct disorder (CD) vs. no psychiatric disorder (NC)

**Social Context Domains**	**CD (n = 52)**	**NC (n = 41)**	**Statistic**	***p***	**ES**^**1**^	**Overall Group Effect *p***
	**Mean (SD) or Percentage**					

**Neighborhood Quality**						0.206

% Vacant houses	10.31 (94.83)	8.50 (5.00)	1.77	0.080	0.37	
% Adults < H.S.^2 ^education	82.83 (7.19)	84.85 (7.44)	-1.32	0.190	-0.28	
% Unemployed adults	59.58 (5.72)	60.70 (5.98)	-0.92	0.359	-0.19	
% Families in poverty	13.04 (7.96)	9.25 (6.41)	2.48	0.015	0.52	
% Single mothers	9.38 (4.53)	7.50 (4.36)	2.01	0.047	0.42	

**Family Characteristics**						0.001
Hollingshead score	48.13 (16.90)	37.22 (16.10)	3.16	0.002	0.66	
Maternal age at girl's birth	23.84 (4.43)	27.10 (5.56)	-3.15	0.002	-0.66	
Married^a^	31	49		0.090	0.42	
Parental ASB^3,a^	69	27		< 0.0001	1.00	
Parental depression^a^	21	22		1.000	-0.03	
Parent SUD^4,a^	35	27		0.502	0.20	

**Parenting Scores**						< 0.0001
Family activities – P^5^	8.55 (4.50)	12.46 (3.96)	-4.08	< 0.0001	-0.92	
Positive discipline – P	5.12 (1.71)	5.12 (1.79)	0.02	0.987	0.00	
Negative discipline – P	14.35 (3.70)	11.36 (3.65)	3.75	0.0002	0.81	
Consistency – P	4.79 (1.24)	5.31 (0.84)	-2.28	0.023	-0.48	
Supervision – P	15.89 (3.29)	17.31 (2.57)	-2.08	0.040	-0.47	
Family activities – Y^6^	7.16 (3.54)	10.08 (3.45)	-3.99	0.0001	-0.83	
Positive discipline – Y	4.33 (2.15)	5.48 (2.12)	-2.57	0.012	-0.54	
Negative discipline – Y	15.10 (4.62)	11.68 (4.20)	3.69	0.0004	0.77	
Consistency – Y	3.86 (1.36)	4.65 (1.86)	-2.37	0.020	-0.49	
Supervision – Y	12.65 (4.41)	15.15 (3.63)	-2.93	0.004	-0.61	

^1 ^Effect size

^2 ^High school

^3 ^CD or Antisocial Personality Disorder

^4 ^Substance Use Disorder

^5 ^Parent report

^6 ^Youth report

^a ^Fisher's Exact Test used

Question 2: Are family characteristics (e.g., family structure and parental psychopathology) correlated with CD?

MANOVA indicated that the social context domain of family characteristics was significantly associated with CD, as is shown in Table 2. For this analysis, we used the total score on the Hollingshead Four Factor Scale for socioeconomic status instead of the five social class categories. After applying a Bonferroni correction for multiple comparisons within the family characteristics domain, higher Hollingshead scores (indicating lower socioeconomic status), lower mean maternal age at daughter's birth, and proportion of parents with antisocial behaviors were still significantly associated with CD.

Group status (CD/NC) was regressed on these variables in a logistic regression and the results are displayed in Table 3. Because of the relatively small sample size, we forced all variables in the equation rather than attempting to build a model. The only variable to remain significantly associated with CD was the proportion of parents with antisocial behavior.

**Table 3:** Logistic regression models predicting conduct disorder:family characteristics and parenting behaviors

**Variables**	***B*(SE)**	**WALD**	**Exp(b)**	**95% CI**	***p *value**
**Family Characteristics**					

Constant	2.57 (1.69)	2.32	13.01		0.128
Hollingshead score	0.02 (.02)	1.19	1.02	0.99 – 1.05	0.275
Maternal age at girl's birth	-.09 (.05)	3.01	0.92	0.83 – 1.01	0.083
Parental ASB^1^	-1.51 (.49)	9.30	0.22	0.08 – 0.58	0.002

**Parenting Behaviors**					

Constant	0.34 (1.25)	0.07	1.40		0.788
Family activities	-.27 (.08)	12.29	0.76	0.66 – 0.89	< .0001
Negative discipline	0.187 (.02)	6.38	1.21	1.04 – 1.39	0.012

Family Characteristics: R^2 ^= 0.22 (Cox & Snell), 0.30 (Nagelkerke), Model X^2^(3) = 23.42

Parenting Behaviors: R^2 ^= 0.27 (Cox & Snell), 0.36 (Nagelkerke), Model X^2^(2) = 28.94

^1 ^CD or Antisocial Personality Disorder

Question 3: Do parenting behaviors differ between girls with and without CD?

Our analysis indicated that the overall social context domain of parenting behaviors was significantly associated with CD (see Table 2). After correcting for multiple comparisons, only parental and youth reports of family activities and negative discipline remained statistically significant. The logistic regression was conducted with family activities and negative discipline as predictors of CD group status. Because parent and youth reports were highly correlated, we used the mean of scores from parent and youth reports on each variable in the logistic regression. Table 3 displays the results of the logistic regression and shows that both variables remained statistically significantly associated with CD.

Question 4: Does race (white or African-American) moderate any of the above associations?

We examined the effects of race interactions with each social context variable entered in the two logistic regression equations. No statistically significant interactions were found.

Question 5: Are the associations between neighborhood or family characteristics and CD in girls mediated by parenting behaviors?

**Figure 1:**
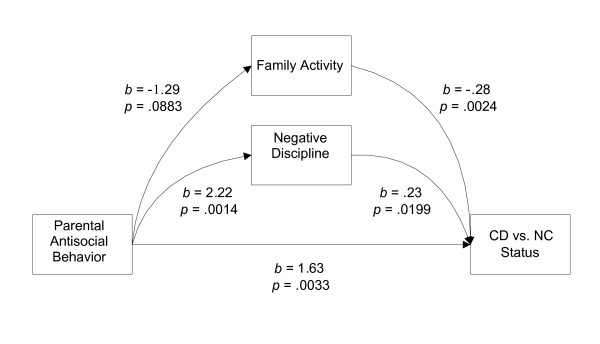
Mediation analysis model.

As presented above, the overall social context level of neighbourhood was not significantly associated with CD. Therefore, we only tested a meditational analysis with the family characteristics domain, using parental antisocial behavior, the only variable that remained significantly associated with CD in the logistic regression analysis. Figure 1 presents the mediation analysis model. All components of the model met Baron and Kenny criteria for a meditational relationship: a) parental antisocial behavior is associated with CD, b) parental antisocial behavior is associated with parenting behaviors and c) parenting behaviors are significantly associated with CD. However, the *z *statistics for the indirect effects of parental antisocial behavior on CD through mediation by parenting behaviors fell short of statistical significance (for the mediating path through family activity, *z *= 1.488, *p *= .137; and for the path through negative discipline, *z *= 1.88, *p *= .060). Thus, our data did not support the hypothesis that the association between parental antisocial behavior and CD in girls could be explained by parenting behaviors.

## Discussion

In this study, we examined three domains of social context in girls with a diagnosis of CD: neighborhood quality, family characteristics, and parenting behaviors. CD in girls was not associated with neighborhood quality, but was correlated with family characteristics and parenting behaviors domains, but there was no moderation by race on these relationships. Although family characteristics (parental antisociality), parenting behaviors, and CD were associated, our hypothesis that parenting behaviors would explain the relationship between parental antisociality and CD was not supported. We improved on previous research by investigating girls with CD who were recruited from the community, thus avoiding the biases that can occur with sampling from clinical or juvenile justice facilities. We also recruited a comparison group that was similar in demographic characteristics.

There are no other studies of neighborhood effects on girls with CD with which to compare our results. However, studies which examine aggression, delinquency, or "problem behaviors" (usually pregnancy, substance abuse, or a sum of symptoms from all the externalizing disorders) and report data specifically on girls, have demonstrated that disadvantaged neighborhood is associated with increased levels of these outcomes [40,41,72,73]. Our findings may have differed because we studied girls who met all the criteria for a diagnosis of CD. Neighborhood quality may have an effect on outcomes such as committing a delinquent act or getting pregnant because these outcomes are more common in girls than is the cluster of specific persistent and pervasive antisocial behaviors known as CD. Neighborhood effects in previous studies could also have been due to the confounding of demographic characteristics with the main outcome variables, a process reduced by our selection of a matched comparison group. This explanation is supported by results of another study in which there was no main effect of neighborhood on elementary school students' aggression (girls and boys) [74]. However, an analysis examining the interactions of race and social class revealed that disadvantaged neighborhood was associated with high levels of aggression, but *only *when the child was black and came from household headed by a single mother. Finally, neighborhood effects on girls with CD may be more complex than we could measure in this study. In the Moving to Opportunity experiment, girls moved to better quality neighborhoods improved more on measures of delinquency, substance use, and risky behaviors than did boys, but this was partially mediated by a decrease in the frequency of peer contacts [75]. Thus, future research on social context in girls with CD should include peer relationships and investigations into a peer relationship-neighborhood quality interaction should be conducted.

Our findings on family characteristics were largely consistent with previous work on CD in girls [33-35] and mixed samples of boys and girls or boys alone [37,76]. Our strong association between parental antisociality and CD in girls support a growing concern that parental antisocial behavior may be particularly problematic for girls, especially if the parent is the mother. Data from another study demonstrated that girls whose mothers had antisocial behavior were 4 times more likely to have CD than those whose mothers did not, even after controlling for paternal sociopathy [77]. We could not dissect out the individual effects of each parent's history on CD because of our sample size, but this issue clearly merits more research.

The absence of significant associations between CD and family characteristics such as social class, maternal age at girl's birth, or marital status is at odds with previous work on girls with CD [33-35]. Because we studied girls who met full criteria for CD and used demographically similar groups, our findings suggest that such family characteristics may not play a primary role in girls' CD. However, we cannot rule out the possibility that our sample was not large enough to detect statistical significance in comparisons with small effect sizes (see Table 2 for our effect sizes). Our study needs to be replicated in a larger sample of CD and non-CD girls before we can conclude that such family characteristics are not associated with CD in girls.

Like results from the earlier studies [33-35], parenting behaviors in our sample were highly correlated with CD. The results were essentially the same for data from both informants. This is consistent with predictions of the social ecology theory by Bronfenbrenner [78]. He proposed that social context domains in which the individual was most intimately involved with others would have more effect on development than domains more distal to the individual (e.g., neighborhood). Girls with CD seem to be more affected by the interactions with parents than with the characteristics of their families or the neighborhoods in which they live. Data from studies comparing girls and boys with problem behaviors or delinquency support this idea and further suggest that problems in interpersonal relationships may be a much more important correlate of girls' CD than male CD [79].

The lack of significant race effects in our study suggests that being white or African-American is not correlated with CD in girls. Race effects have been reported in some studies of problem behaviors or delinquency in mixed samples of boys and girls [50,80], but not in others [52]. Rates of delinquency in the U.S. are higher in African-American girls than in the white population [81], but this is likely due to social bias in the arrest process.

Our lack of significant support for our meditational model indicates that family characteristics (parental antisociality) and parenting behaviors (levels of family activities and negative discipline) are associated with CD in girls through mechanisms different than the ones we proposed. Rhule and colleagues also obtained null results with a model similar to ours in a longitudinal study of mothers' antisocial behavior and externalizing disorder symptoms in their third-grade children [82]. Longitudinal data are needed to better understand the mechanisms of how these social context domains are linked in girls with CD, but it is possible that parental antisocial behaviors affect girls' CD through genetic pathways or behavioral modeling, particularly if it is the mother who is antisocial. Negative discipline and lack of family activities are clearly important factors in girls' CD, but our hypothesized model may not be valid because such parenting problems are not be specific to parental antisociality. For example, children of chronically depressed mothers have high rates of antisocial behaviors. Interestingly, this effect is larger in girls and the effect is increased if one or both of the parents also have antisocial behavior [83-85].

There are important limitations to our study. The first is sample size. Our sample contained the same, or more, girls with the diagnosis of CD than the three previous studies, but it still may not have been sufficiently large to detect statistically significant small effect sizes. Therefore, any null findings should be interpreted with caution. Second, we conducted a cross-sectional study. This limits our ability to draw conclusions about how social context affects the development of CD in girls. Third, we did not assess reciprocality in our study, a process important at each social context level and one that has recently been demonstrated as a potential mechanism explaining parenting behaviors and CD symptoms in pre-adolescent girls [86]. Our parenting behavior data were collected from questions with a unidirectional perspective (parent to girl), but when interpreting our findings, it should be kept in mind that parenting behaviors are often affected by the youth's behavior.

## Conclusion

Our results have implications for treatment and future research. The traditional psychiatric examination of a girl with CD collects some family history and social data, but the main focus is on the girl's behaviors and symptoms. We suggest that clinicians broaden their evaluations to include assessment of parental psychopathology and parenting behaviors, particularly the level of family activities and the types and frequency of negative discipline techniques. Treatment may be more successful if parents are included.

Our correlational study of three social context domains should be viewed as hypothesis generating. We have three recommendations to improve future research on social context and CD in girls. First, peer networks and school relationships should be added to the social context domains examined in our study. Second, larger samples of girls with CD and demographically matched comparison girls should be used. This will enable researchers to take advantage of more sophisticated analytic approaches (e.g., structural equation modelling). Third, researchers should explore models that incorporate associations between domains and include multiple indicator variables for each domain.

## Competing interests

The authors declare that they have no competing interests.

## Authors' contributions

KP designed the study, obtained funding for it, collected the data, and wrote or edited all drafts of the manuscripts. SS coded and entered the data and conducted the literature review, writing the first draft of the Background, Results, and Discussion, with the assistance of KT and CC. WG and WW were responsible for the statistical analyses and edited relevant sections of the manuscript. All authors read and approved the final manuscript.
